# A sequence based synteny map between soybean and *Arabidopsis thaliana*

**DOI:** 10.1186/1471-2164-8-8

**Published:** 2007-01-08

**Authors:** Jeffry L Shultz, Jeffery D Ray, David A Lightfoot

**Affiliations:** 1 USDA-ARS, Crop Genetics and Production Research Unit, P.O. Box 345, Stoneville, MS 38776, USA; 2 Dept. Of Plant Soil and Agricultural Systems, Genomics and Biotechnology Core Facility, Center for Excellence in Soybean Research, Southern Illinois University, Carbondale IL, 62901, USA

## Abstract

**Background:**

Soybean (*Glycine max*, L. Merr.) is one of the world's most important crops, however, its complete genomic sequence has yet to be determined. Nonetheless, a large body of sequence information exists, particularly in the form of expressed sequence tags (ESTs). Herein, we report the use of the model organism *Arabidopsis thaliana *(thale cress) for which the entire genomic sequence is available as a framework to align thousands of short soybean sequences.

**Results:**

A series of JAVA-based programs were created that processed and compared 341,619 soybean DNA sequences against *A. thaliana *chromosomal DNA. *A. thaliana *DNA was probed for short, exact matches (15 bp) to each soybean sequence, and then checked for the number of additional 7 bp matches in the adjacent 400 bp region. The position of these matches was used to order soybean sequences in relation to the *A. thaliana *genome.

**Conclusion:**

Reported associations between soybean sequences and *A. thaliana *were within a 95% confidence interval of e^-30 ^– e^-100^. In addition, the clustering of soybean expressed sequence tags (ESTs) based on *A. thaliana *sequence was accurate enough to identify potential single nucleotide polymorphisms (SNPs) within the soybean sequence clusters. An EST, bacterial artificial chromosome (BAC) end sequence and marker amplicon sequence synteny map of soybean and *A. thaliana *is presented. In addition, all JAVA programs used to create this map are available upon request and on the WEB.

## Background

Recent soybean genetic maps have used combinations of classical and molecular markers to determine the approximate order of sequences on soybean chromosomes viewed as linkage groups [1-9]. The newest versions of the integrated soybean genetic map [7] and physical map [10,11] provide milestones in soybean genome map development. Methods used in creating these map resources have led to the creation of a large number of discrete sequences from BAC-ends and marker amplicons. All of these sequences identify either a contig of clustered sequences located in a physical map or a single mapped location in a genetic map. The largest number of soybean sequences available are expressed sequence tags (ESTs), which were created during public soybean EST projects [12] as well as in numerous other soybean research projects. These sequences represent fragments of DNA that are expressed as mRNA constitutively or during environmental changes. A public repository for all of these sequences is the National Center for Biotechnology Information [13]

Although soybean map resources are advanced, they cannot compare to the information available from fully sequenced genomes [14]. Therefore, comparisons among map and sequence resources of nascent genomes with the elucidated pathways or markers in *A. thaliana *is an appropriate and informative technique [15]. A syntenic relationship between *A. thaliana *and *Glycine max *L. (Merr.) based on restriction fragment length polymorphism (RFLP) marker order has previously been established [16,17]. Additional soybean sequence resources have become available [10], and a draft genome is promised, making large-scale sequence-based comparisons with *A. thaliana *possible and timely.

EST resources can be used for genome to genome comparisons. For example, a comparison of soybean ESTs with those from corn, rice, sorghum, barley, potato, tomato and *Medicago truncatula *[18] focused on evolutionary distance and synteny between ESTs. In addition, soybean ESTs have been compared with lupin (*Lupinus angustifolius *L.) and *A. thaliana *[19], detailing gene structure and expression. Molecular markers have been developed for soybean using comparison with the closely related *Lotus japonicus *genome [20]. The *A. thaliana *Diacylglycerol acyltransferase (DGAT) gene sequence was used to identify putative *G. soja *and *G. max *EST sequences [21]. Additional comparative genomics resources are available at the Legume Information System (LIS) website [22], but do not show the placement of soybean sequences *en-masse *against *A. thaliana *genomic sequence.

Synteny between genomes shown by common molecular marker order(s) suggest common genome structures. [20,22]. Comparisons among tracts of sequence show precise sequence synteny [19,21]. Orthologous and paralogous relationships between small regions of soybean, *Medicago truncatula *and *A. thaliana *has been reported [23]. Finally, comparisons of changes within and between sequences are evidence of evolutionary change and selective gene loss [18].

The first objective of this research was to develop a program for large scale DNA sequence comparisons, utilizing large tracts of well described genomic DNA (*A. thaliana*) as a framework to align thousands of short soybean sequences. The second objective was to present these comparison results in an informative form. In this report we describe the development and operation of programs designed to compare genomic sequence with smaller fragments and the creation of genome-wide illustrations of sequence synteny.

## Results and discussion

### Short soybean sequences compared with A. thaliana

The importance, variety and number of short soybean sequences and the availability of well described *A. thaliana *genomic sequence were the primary factors involved in selection for this test. On average, the program compared over 160 Tbp/hr ((3,000 soybean cDNA records with an average of 461 bp each) × 119 Mbp of arabidopsis DNA) = 164.5 Tbp/hr). A total of 33,106 short soybean sequence matches with *A. thaliana *were reported from 341,619 unique records (9.7%). Of these matches, a total of 30,014 soybean ESTs (about 3,000 unique genes), 2,946 BES, 135 microsatellite, 6 FiS sequences, and 5 GmClone associations were reported. This association of genes and sequences between soybean and *A. thaliana *can serve several functions, including identification of gene-clusters within soybean, identification of potentially syntenic chromosomal regions, soybean expression estimation and single nucleotide polymorphism detection within soybean sequences.

Theoretically, a random 15 base pair match should occur every 1 Bbp. Previous work (unpublished) and the fact that the DNA sequence tested was primarily not random (i.e. ESTs) led to the multiple, local 7 bp match requirement. Each 7 bp (p = 1e^-05^) match was found within a 400 base pair region, and empirical analysis was used to determine that six of these additional matches were a practical minimum. In order to test the accuracy of the soybean vs *A. thaliana *matches, e-scores for 160 randomly distributed matches from each *A. thaliana *chromosome were generated by using NCBI's BLAST 2 Sequences (bl2seq) utility. As Figure 2 illustrates, increasing the number of additional 7 bp hits does increase the average e-score when comparing sequences from these two genomes. The number of additional 7 bp hits can be used post-processing, allowing the user to investigate the parameters required for acceptable similarity while only running the program once.

The five separate chromosomes of *A. thaliana *were put into Mapchart [24] format for presentation. Reported matches to each chromosome are presented in color, linear order maps (supplemental; B&W Figure 3). Genic regions reported for *A. thaliana *(30,694) are identified by dark (blue) segments and light (blue) descriptive text. Soybean EST and BES sequences are both in black text, with soybean markers presented in red.

### Comparison with previously reported soybean-A. thaliana synteny

As previously noted, marker synteny between *A. thaliana *chromosome I and soybean linkage group A2 (including homeologous regions of A1, E and C2); and IV and A2 (including II/IV and J/L homeology) has been reported [17]. The inclusion of RFLP and simple sequence repeat (SSR) amplicon data from soybean allows limited comparison with this previous work. Although sequences from SSR amplicons did match to *A. thaliana *DNA sequence using this procedure, SSR sequences by definition contain repeats and usually yielded non-significant results when tested by the BL2SEQ utility [17]. When SSR containing BACs were analyzed (denoted in the map as a BAC plate location followed by SSR and soybean chromosome location), significance was similar to SSR amplicons, i.e. limited. Although both of these data types are included in the map, they were not as reliable as EST-based matches. It must also be noted that gene order between *A. thaliana *and soybean is not inferred by this research, only gene content.

An illustration of the effects of sequence size and comparison power was found in the comparison of the Satt238 amplicon (435 bp) with *A. thaliana *chromosome I (30,432,563 bp). The program reported a (23 bp) match at base pair 961,168. A comparison of the entire *A. thaliana *chromosome I (NC_003070) and this sequence (gi14969942) yielded no significant similarity. However, using a 2000 bp window of AtI DNA (960,000–962,000 bp) yielded an expect value of 1.0. Decreasing the *A. thaliana *DNA window to 961,150–961,190 bp yielded a 0.009 significance level. Finally, reducing the sequence to 961,163–961,185 yields a 0.001 result. Although previous analysis [17] was performed using tBLASTx, this type of analysis yielded no significant results for any of the above marker sequence comparisons.

Comparing the soybean gi sequences displayed in Figure 3b with the reported *A. thaliana *chromosome V region (15,426,000–15,429,000) yields four linear order groups. The first group, gi15200550–6913885, was similar to *A. thaliana *chromosome V (range e^-67 ^– e^-87^), e-values between the soybean sequences are 0.0. The second group, gi10237097–19054121, was similar to chromosome V (range e^-60 ^– e^-83^), e-values between the soybean sequences are e^-112 ^– 0.0. The third group, gi16347707–4306793, was similar to chromosome V (range e^-06 ^– e^-15^), e-values between the soybean sequences are e^-163 ^– 0.0. The fourth sequence, gi5676855, was similar to chromosome V at e^-20^. The identification and clustering of similar soybean sequences based on their position on *A. thaliana *DNA was a result of this technique. Soybean sequences that grouped together frequently had bl2seq scores of 0.0, with values rarely above e^-100^. The ability to detect SNPs within these grouped soybean sequences is illustrated in Figure 3c.

### Critical program parameters

The ability to start each check at an initial 5 bp identity increases processing speed by nearly 1000×. A short sequence advance value determines whether each 15 base pair fragment is advanced by one, two, or more bases for each screen of the model (*A. thaliana*) DNA sequence, with the potential of increasing the speed by reducing the number of sequence screens.

Although all soybean sequences were sequentially placed in relation to the *A. thaliana *genome, a limitation on map precision means that separation into uniquely delimited map entries does not occur within a 400 bp range. For example, in Figure 3b, a unique callout containing gi #s 15200550 thru 4306793 is presented. A gap of 124 bp existed between gi6913885 (15,427,045 bp) and gi10237097 (15,427,169 bp). In addition, a gap of 244 bp between gi19054121 (15,427,172 bp) and gi16347707 (15,427,416 bp) also exists. These gaps may represent weaker than typical links between grouped soybean records (although still significant matches at 4 e^-158 ^and 4 e^-18 ^respectively).

## Conclusion

We report an alternative technique for large scale DNA sequence comparison and the creation of an *A. thaliana*-based map of soybean ESTs, BAC-end and molecular marker sequences. This map is comprised of 63 highly detailed, fully scalable, color-coded sub-maps. These maps allow the rapid, visual identification of clusters of both high and low quantities of transcribed soybean ESTs and their relation to *A. thaliana *genomic sequence. To our knowledge, no similar tool exists.

The applications of this processing technique and resulting map appear to be broad. As previously indicated, EST-based comparisons between soybean and disparate genomes have been used to explore evolutionary distance and synteny [18]. The process presented herein utilizes small fragments of DNA and allows users to increase or decrease the similarity required for whole genome comparisons. The flexibility of smaller fragment comparisons allows the creation of highly rigorous comparative maps or less stringent, theoretical maps from diverse sources. Most importantly, these maps can be created from thousands of discrete sequences with little *a priori *knowledge of genome composition. Indeed, a model genome can be used to cluster sequences from any source(s), straight from downloaded FASTA formatted files.

A limitation when utilizing a model legume and comparative mapping for soybean marker development was reported by Hwang et al [20]. This limitation relates to the multi-copy nature of many genes in soybean and the difficulty of separating these homeologous genes in the EST dataset. Mapping these genes can be difficult when homeologous sequence confounds PCR-based allele identification. The identification of sequence differences between homeologous genes from the same variety may in fact be the only way to specifically map homeologous genes to the correct linkage group of soybean. The clustering of soybean ESTs shown on the map presented offers unique visual clues as to copy number and expression characteristics of each gene, both within and between soybean varieties.

Previously created comparative maps between soybean and *A. thaliana *relate only a handful of ESTs or RFLPs between the two genomes at cM-sized intervals [17,20]. Because our map is based on the entirety of the *A. thaliana *genome, it identifies not only regions reported as syntenic, but regions that are potentially syntenic, In addition, our map exhibits previously unreported detail, reporting differences as low as 400 bp. The combination of previously reported synteny between *A. thaliana *and soybean and the detail of this map combine to make this map a powerful *exploratory *tool.

The variable nature of output from this processing has clear implications for genetic and physical map construction and annotation of either large fragments of DNA or thousands of smaller fragments such as ESTs and BAC-end sequences. This technique reports significant matches when performing comparisons between small fragments of DNA and large genomic tracts which is an advantage over tBLASTn-based analysis. This technique and the programs presented readily allow sequence comparisons across genera. Finally, the programs required to perform this analysis require limited computing resources, specifically a computer capable of running JAVA programs and a minimum of 1 GB of memory. All JAVA code and map files are available for download at two websites [25,26].

## Methods

### Sequences used for genome comparison

A total of 326,677 soybean cDNA records were downloaded from NCBI in FASTA format. The records contain 150,810,754 bp of sequence, with an average of 461 bp per record. In addition, there were 13,472 BAC-end sequences (9,955,983 bp) with an average of 738 base pairs per record. These were largely unique (about 9.96 Mbp) but predominantly non-coding (about 8.1 Mbp) since they were from a minimum tile [10]. Minor sequences used were 936 simple sequence repeat (aka microsatellite; hereafter SSR) marker amplicons (489,077 bp), 77 FiS (subtraction library of 'Forrest' infected with *Fusarium solani*) sequences (36,066 bp) and 457 restriction fragment length polymorphism (RFLP) sequences (304,508 bp). In total 161,596,388 base pairs of soybean DNA in 341,619 fragments were downloaded from NCBI and included in this analysis. The five chromosomes of *A. thaliana *contain 119,186,014 base pairs. The *A. thaliana *DNA sequence was downloaded from NCBI in FASTA format (NC_003070, NC_003071, NC_003074, NC_003075, NC_003076) and used as the framework to align the soybean sequences.

### Sequence similarity program

In brief, three processes were used to create the sequence synteny map. The first process was the preparation of sequences, during which *A. thaliana *DNA was placed into a single linear sequence and then screened for unique five base sequences. The second process was the comparison of soybean sequences against the prepared *A. thaliana *linear sequence. Finally, information about match locations and unique identifiers (gi numbers/clone ID) were merged with *A. thaliana *gene information to form the map.

During sequence preparation, FASTA formatted reference sequence (*A. thaliana*) was processed to remove all line feeds, headings or other special characters and place the DNA in uninterrupted linear order from chromosomes I to V. Secondly, the same program indexed this modified file for 5 base sequences by advancing through the sequence 1 base pair at a time. As each 5 base sequence was encountered, the next two bases in the sequence were added. In order to reduce repeat DNA matches (i.e. attatt, atatat, aaaa, etc) each base (A, T, G and C) was required in this 5+2 base sequence. If all four bases were present, the location was written to a 5 base sequence array of 1020 possible combinations. This indexing created an array of 5 bp sequence locations with reduced repeat representation specific to *A. thaliana*.

**Figure 1:**
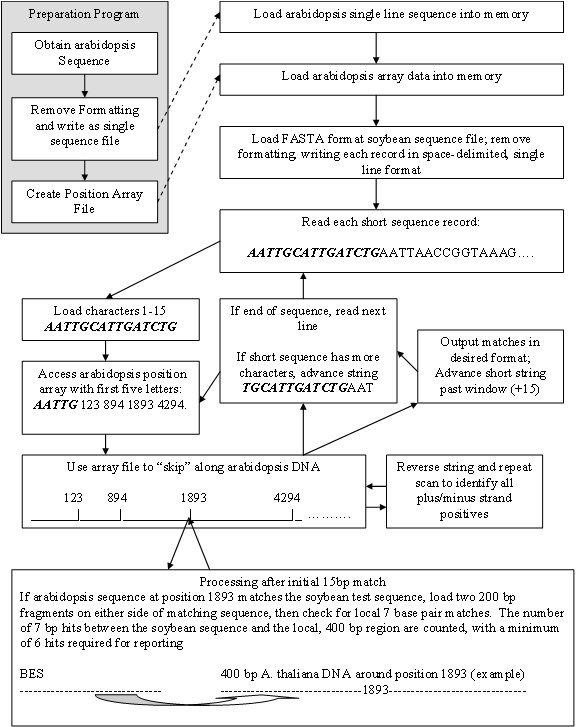
General program flow diagram for comparison of short (<1,000 bp) sequences against a model genome. In this example, soybean EST, BES, RFLP and SSR amplicon sequence is compared to the five chromosomes of *A. thaliana*.

**Figure 2:**
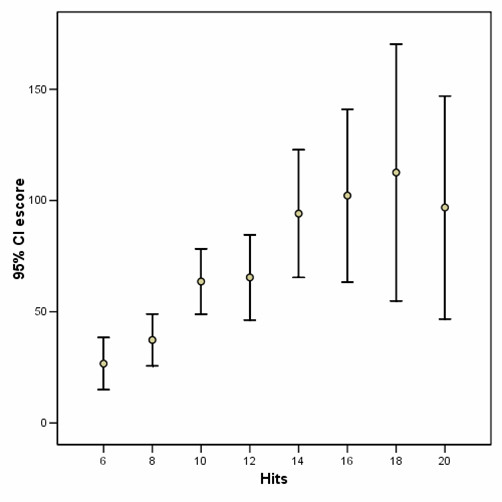
Confidence intervals for expect (e) values between soybean sequences and *A. thaliana *genomic sequence, based on the number of 7 bp hits in addition to an initial 15 bp identity (SPSS Inc., Chicago, Illinois). For each category, a total of twenty, randomly selected matches sharing the same number of additional, identical 7 bp hits were compared using the BLAST2Seq utility on NCBI. The e-score values reported are those between the soybean sequences and the *A. thaliana *chromosome region indicated by the BLAST2seq program. For instance, when 10 additional 7 bp matches are identified by our algorithm, 95% of the short soybean sequences identified as similar to an *A. thaliana *genomic region had e values between e-^50 ^and e-^80 ^when these two sequences were compared on NCBI.

**Figure 3:**
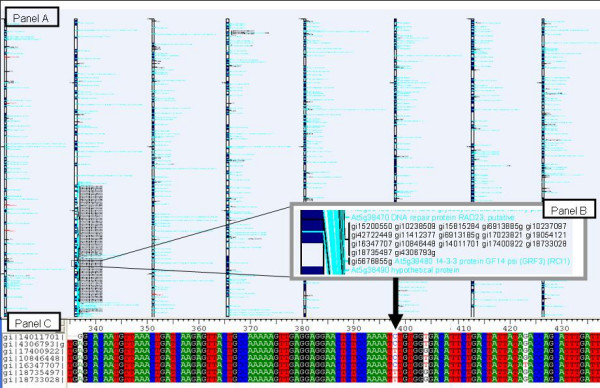
Panel (A) A Synteny map of soybean sequences compared to *A. thaliana *genomic sequence. The region displayed is from *A. thaliana *chromosome V, 15,000,000 – 17,250,000 bp. Solid chromosomal segments represent reported *A. thaliana *genes, described in light text. All "gi" features are soybean ESTs, All "H" or "B" features represent bacterial artificial chromosome (BAC) end sequence. Map display created in MapChart and exported in enhanced metafile format. Each vertical segment is equivalent to 250,000 bp of *A. thaliana *DNA. Panel (B) is an expanded view of the 15,400,000 to 15,500,000 bp region. Panel (C) is a Clustal alignment of soybean ESTs (gi #'s) listed in Panel b. Varieties listed are: gi14011701 *(Williams 82)*; gi4306793 (*Williams*); gi17400922 *(PI 567374)*; gi10846448 *(Williams 82)*; gi16347707 *(Williams)*; gi18735497 *(Jack)*; gi18733028 *(Raiden)*. Single nucleotide polymorphisms are apparent at base pair locations 337, 398, 404 and 423 in panel C.

The comparison step was performed by loading the array of 5 bp *A. thaliana *sequence positions into the next program, followed by the *A. thaliana *nucleotide-only sequence. FASTA formatted soybean sequences (EST, BES, SSR, etc) were then read into the program, with each sequence being screened against the *A. thaliana *sequence (Figure 1). In brief, a five base nucleotide sequence from the short sequence was used to access the array of 5 bp sequence locations, allowing the program to check only those sequences that shared the same first five base pairs. This process allowed the program to "skip" along the *A. thaliana *DNA sequence. If a 15 base match was subsequently identified, it was followed by local, 7 bp sequence comparisons using a 400 bp fragment generated from 200 bp up- and downstream sequences. If at least 6 additional, random order, 7 bp matches were found, the location was divided by 10,000, assigned to a chromosome of *A. thaliana *based on this number, output with four decimal places, given the appropriate soybean ID, with additional annotation of the number of additional 7 bp matches. Each short sequence was reversed and the process was repeated.

Execution times for the 341,619 (161 Mbp) short soybean sequences compared with the 119 Mbp of *A. thaliana *DNA sequence were approximately 3,000 short sequences per hour (RedHat Linux o/s, 2.2 GHz processor, 1 GB RAM). All programs were written in JAVA, allowing compatibility with any operating system capable of executing JAVA programs. After processing was complete, the Blast2 sequences program 27 was used to confirm random and selected matches. All sequences were compared using the default settings on the NCBI website. Confidence intervals for e values from 20 randomly selected *A. thaliana *and soybean matches with 6, 8, 10, 12, 14, 16, 18 and 20 additional 7 bp matches were calculated using the SPSS statistical program (SPSS Inc., Chicago, Illinois)

Synteny maps were prepared for Mapchart [24] presentation. Soybean sequence matches were sorted based on *A. thaliana *base pair location. Shaded areas depicting genic regions in *A. thaliana *were created by using the "segments" data type in Mapchart. All maps were exported in enhanced metafile format, viewable as color, completely scalable Microsoft Windows™ Picture and Fax viewer files.

## Abbreviations

BAC, bacterial artificial chromosome; bp, base pair; EST, expressed sequence tag; LIS, legume information system; NCBI, National Center for Biotechnology Information; RFLP, restriction fragment length polymorphism; SNP, single nucleotide polymorphism; SSR, simple sequence repeat.

## Authors' contributions

JS conceived of the study, created and executed all programs and drafted the manuscript. JR and DL provided critical review, interpretation of results and funding. All authors read and approved the final manuscript.
